# Soluble expression of recombinant active cellulase in *E.coli* using *B.subtilis* (natto strain) cellulase gene

**DOI:** 10.1186/s43141-020-00103-0

**Published:** 2021-01-11

**Authors:** Bhuvan Shankar Vadala, Sumedh Deshpande, Anjali Apte-Deshpande

**Affiliations:** Central Dogma Pvt. Ltd, A4, Gulmohar Residency, Baner Road, Baner, Pune, Maharashtra 411045 India

**Keywords:** Cellulase, Recombinant expression, *B. subtilis*, pET21a

## Abstract

**Background:**

Cellulases are well known for their various industrial applications. They are naturally produced by different species of bacteria and fungi. Fermentation process of cellulase producers has limitation due to the high substrate cost required for cellulase induction and challenges to maintain the suitable condition for the respective cellulase production. Recombinant cellulase production could be the potential solution to these problems. In the current study, we investigated recombinant cellulase expression in *Escherichia coli* using cellulase gene from *Bacillus subtilis*.

**Results:**

Extracellular cellulase production from *B. subtilis* strain was first confirmed on CMC agar and then the cellulase gene (1500 bp) was amplified from this strain and was further cloned in pET21a expression vector. In initial experimental studies, recombinant cellulase expression was achieved in inclusion bodies through shake flask level fermentation of transformed *E. coli* expression host BL21DE3. Attempts were made to express this 55 KDa His tagged recombinant cellulase into soluble form by modifications in fermentation conditions. Partially purified recombinant cellulase was obtained using Ni-NTA affinity chromatography. The activity of the purified enzyme was confirmed by 3,5-dinitrosalicylic acid (DNS) qualitative assay.

**Conclusion:**

Soluble expression of active recombinant cellulase can be achieved by subtle alteration in the upstream process.

## Background

Cellulases have a wide range of industrial applications in textile, pulp and paper, brewery and wine extraction, animal feed industries, along with agriculture [1]. The major industrial application of cellulases is in the textile industry to produce the stone-washed look of denims and for the household laundry detergents for refining fabric softness and brightness [2].

Cellulases are produced in large scale using fungi, bacteria or actinomycetes. The high cost of cellulases is mainly due to the substrates used in the production, and slow growth rate of fungi [1]. There are several reports on production of cellulases from bacteria such as *Bacillus* [3], *Clostridium* and *Ruminococcus* [4] and *Streptomyces* spp., [5]. Microbial cellulases consist of three types of enzymatic activities such as (1) Endo-(1,4)-β-D-glucanase, (2) Exo-(1,4)-β-D-glucanase and (3) β-glucosidase [6]. These enzymes can either be in single subunit form, particularly in aerobic microorganisms, or grouped in a multicomponent enzyme complex called ‘cellulosomes’ in anaerobic cellulolytic bacteria [7].

*Bacillus* spp. is highly studied for its diverse range of cellulases that are stable under extreme conditions [8, 9]. Amongst all *Bacillus* spp., *Bacillus subtilis* continues to be a dominant workhorse due to its capacity to secrete large quantities of extracellular cellulolytic enzymes [3, 10–12]. Many *B. subtilis* strains showing cellulase activity have been isolated from various sources like soil irrigated with paper and pulp mill effluent [13], gut of termites [14], alkaline soil sample [15] and even from unexplored niches. It is been reported that *B. subtilis* (natto strain) which is used to prepare fermented soybean product called natto for several years produces multiple enzymes and one of them is cellulase [16]. There are not many reports about the cellulase gene from this strain which is mainly used as probiotic.

Cellulase production is possible in two ways, one from naturally producing microorganism and second with recombinant expression in prokaryotic or eukaryotic expression system. The naturally produced cellulases are functionally active but get produced in low concentrations, the reason being the conditions for enzyme production are probably not always favourable. Also, these cellulases require specific type of substrates, for which availability and cost both are important criteria. Hence, researchers have focused on developing recombinant cellulase using *Escherichia coli* expression system. *Escherichia coli*, as a host, does not require specific media and grows robustly. The major advantage of recombinant cellulase is the scaleup at the production level without the use of expensive substrate [5, 8, 10, 12–14, 17, 18].

Here, we report on the cloning of the cellulase gene from *Bacillus subtilis* (natto strain) which was isolated from the Japanese food, natto, in T7 promoter-based vector (pET21a). We have optimised fermentation conditions for soluble expression of recombinant cellulase (Endo-(1,4)-β-D-glucanase) in *E.coli* expression host and the enzyme was further purified by metal affinity chromatography. The activity of recombinant cellulase was evaluated by 3,5-dinitrosalicylic acid (DNS) assay [19].

## Methods

### Bacterial culture and chemicals

*Bacillus subtilis* (natto CGMCC2108 strain), gifted by one of the collaborators during research work. pET21a vector, *E. coli* strains BL21 (DE3) and DH5α were purchased from Novagen (USA). Nickel-nitrilotriacetic acid (Ni-NTA) matrix for affinity chromatography was procured from Qiagen (Germany).

### Primer designing

Oligonucleotides were designed with suitable restriction enzyme sites and 6X Histidine (His) tag. Forward primer is with a *Bam*HI site and His tag 5′ CCG GGA TCC CAT CAT CAT CAT CAT CAT ATG AAA CGG TCA ATC 3′ and the reverse primer is with *Eco*RI and *Hind*III site 5′ CCG GAA TTC AAG CTT CTA ATT TGG TTC TGT TCC TCA 3′. Primers were synthesised by Sigma (USA).

### Gene amplification and cloning

Available strain of *B. subtilis* was tested for cellulase activity on carboxymethyl cellulose (CMC) agar and then cellulase gene was amplified from its genomic DNA using forward and reverse primers mentioned earlier in a 25-μL reaction volume. Two-step polymerase chain reaction (PCR) programme was set up for amplification with initial denaturation at 94 °C for 7 min followed by 5 cycles of denaturation at 94 °C for 45 s, annealing at 47 °C for 45 s, elongation at 72 °C for 90 s. Later, 30 cycles were denaturation at 94 °C for 45 s, annealing at 61 °C for 45 s and elongation at 72 °C for 90 s. The resultant amplicon of 1500 bp was checked on 1% agarose gel. It was then purified using PCR purification kit and digested with ‘*Bam*HI’ and ‘*Hind*III’. After purification of the digested amplicon, it was ligated into the pET21a vector double digested with the same enzymes. This ligation mix was transformed into *E. coli* (DH5α) competent cells and recombinant clone was confirmed by colony PCR using gene-specific primers.

### Shake flask level fermentation and optimisation of fermentation conditions

pET21a-cellulase recombinant plasmid was transformed into *E. coli* BL21 (DE3) competent cells. For expression, the culture was grown in LB broth with 100 μg/ml final concentration of ampicillin till it reaches 0.8-1 OD. Further, the culture was induced with isopropyl β-D-1-thiogalactopyranoside (IPTG) 1 mM final concentration and incubated at 37 °C and 180 rpm in a shaker incubator for 4 h. At the end of the induction period, the cells were harvested and lysed by mechanical lysis (glass beads) method for protein extraction. Expression of protein was confirmed on 10% sodium dodecyl sulphate polyacrylamide gel electrophoresis (SDS-PAGE).

For optimisation of soluble protein expression, the induction concentration of IPTG was reduced (0.5 mM final concentration) and the induction time was increased to 12 to 16 h at 28 °C. Protein profile was checked on 10% SDS-PAGE.

### Protein purification by metal affinity chromatography

Supernatant of the cell lysate was passed through Ni-NTA matrix to purify His tagged cellulase by metal affinity chromatography. Matrix was saturated with binding buffer/wash buffer (300 mM NaCl, 10 mM Tris Cl pH 8). After saturation, the supernatant of cell lysate was loaded. Loosely bound contaminant proteins were washed with wash buffer containing 20 mM imidazole. After matrix washing, elution was carried out using an imidazole gradient (250 mM and 500 mM). The protein purity of the eluted fraction was checked on 10% SDS-PAGE.

### Cellulase enzyme activity

Qualitative analysis of recombinant cellulase was performed by DNS assay. Reaction was set up as 0.45 ml 1% CMC in 0.1 M sodium citrate buffer (pH 5) mixed with 0.05 ml enzyme source (crude lysate as well as purified elution fraction in separate tubes). Blank reaction was set up replacing enzyme source with distilled water. Sample and blank tubes were incubated at 55 °C for 30 min. Post incubation, 0.5 ml of DNS reagent was added to all the tubes and kept on a boiling water bath for 5 min. Development of orange colour indicated the generation of free glucose residues post digestion of substrate with cellulase.

## Results

### Inherent cellulase activity of *Bacillus subtilis* (natto CGMCC2108 strain)

**Fig. 1:**
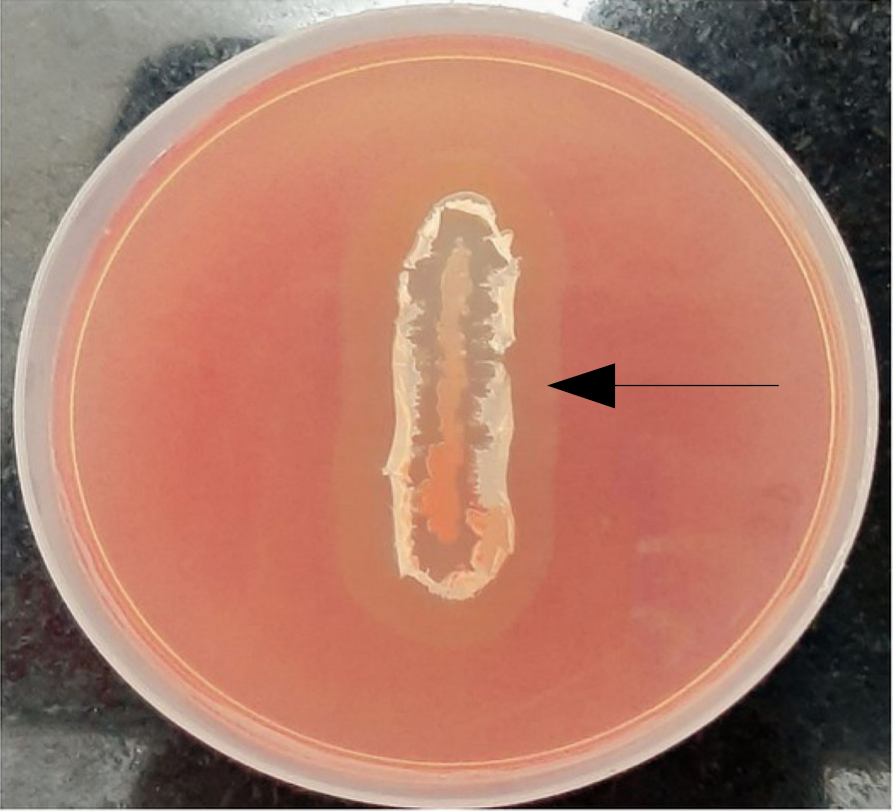
Cellulase activity by *B. subtilis*: *B. subtilis* culture when grown on CMC agar gives zone of clearance due to cellulase production

Cellulase activity was ensured before cloning and recombinant expression in the bacterial system. As is evident in Fig. 1, the zone of clearance surrounding the bacterial growth confirms the production of cellulase enzyme by the strain.

### Cloning of cellulase gene in pET21a vector

**Fig. 2:**
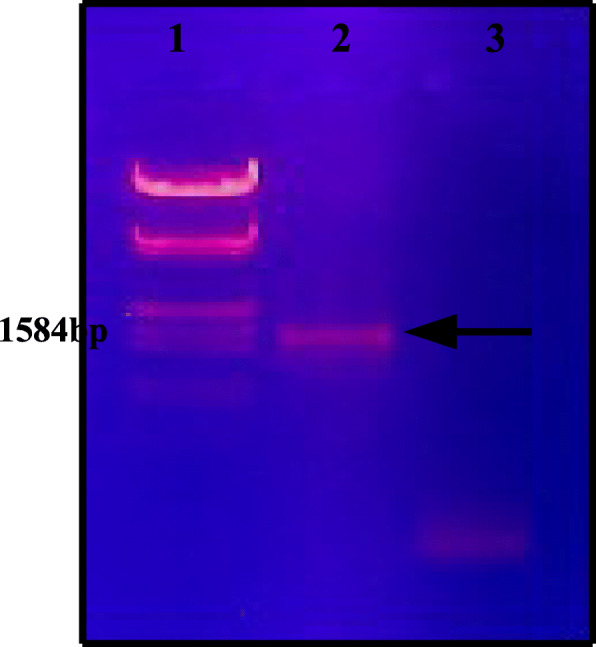
PCR amplification of cellulase gene from *B. subtilis* genome on 1% agarose gel. Lane 1, λDNA *Eco*RI*/Hind*III marker (Thermo Scientific); lane 2, cellulase amplicon; lane 3, non-template control

The cellulase gene was PCR amplified from *Bacillus subtilis* genomic DNA template to PCR product of 1500 bp was confirmed by loading on 1% agarose gel (Fig. 2) along with standard DNA marker (*Eco*RI *and Hind*III digested λ DNA). Amplified gene was digested with ‘*Bam*HI’ and ‘*Hind*III’ and ligated into the pET21a vector digested with ‘*Bam*HI’ and ‘*Hind*III’ to obtain the recombinant.

### Confirmation of clone by colony PCR

**Fig. 3:**
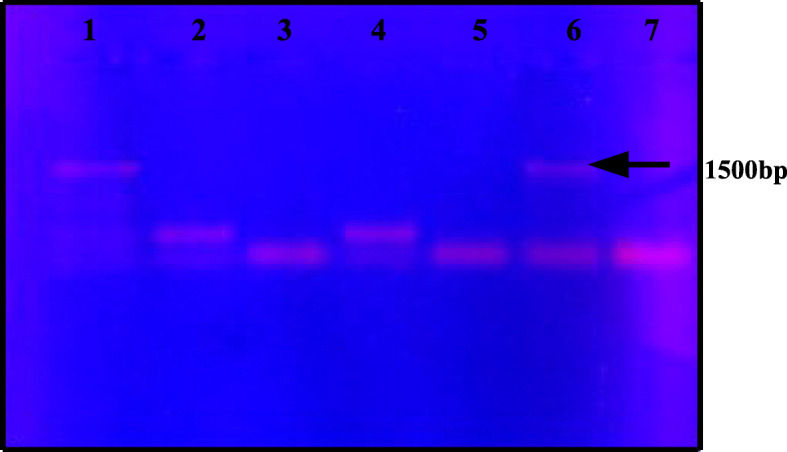
Screening of recombinants by colony PCR on 1% agarose gel. Lane 1, positive control (amplicon from genomic DNA); lane 2-6, colony PCR products; lane 6, size of amplicon matching positive control (pET21a-cellulase recombinant); lane 7, colony carrying pET vector without gene (negative control)

The recombinant clone of pET21a cellulase was confirmed by colony PCR using gene-specific primers. Amplicon size was compared with the positive control (amplicon where *Bacillus subtilis* genomic DNA was used as a template) on 1% agarose gel. As is evident in Fig. 3, out of 9 colonies screened, colony 6 showed an expected size of amplicon.

### Recombinant cellulase expression

**Fig. 4:**
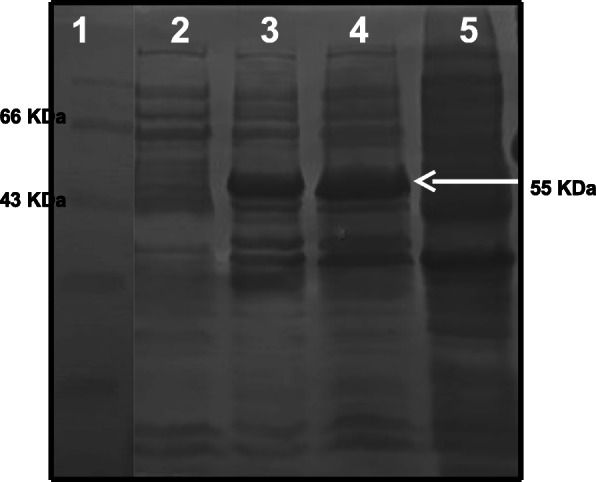
Analysis of expressed recombinant cellulase on 10% SDS-PAGE. Lane 1, protein marker (GeNei); lane 2, supernatant of cell lysate; lane 3, pellet of cell lysate (20 μl); lane 4, pellet of cell lysate (40 μl) with arrow showing expressed cellulase protein; lane 5, BL21 cells carrying pET21a plasmid without gene

pET21a-cellulase clone was transformed into *E. coli* expression host BL21 (DE3). Culture induced with IPTG was analysed on 10% SDS-PAGE for overexpression of recombinant cellulase (Fig. 4) with reference to the protein marker (medium range).

### Optimisation of fermentation conditions

**Fig. 5:**
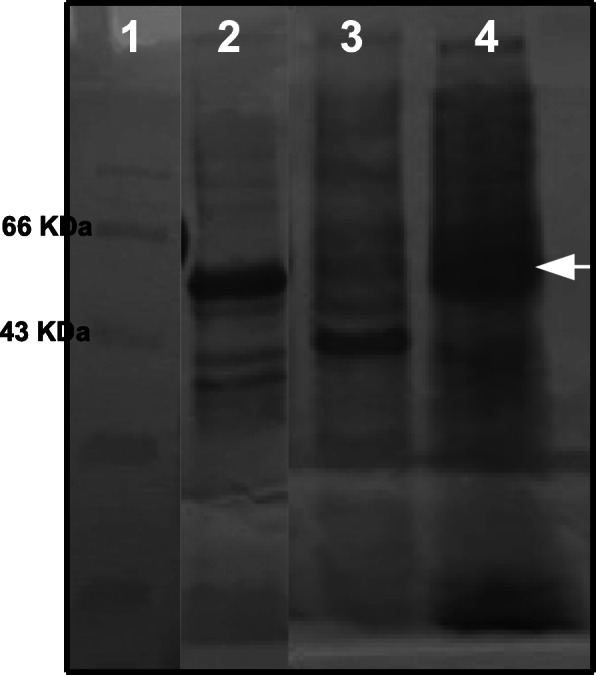
Cellulase expression with increased solubility. Lane 1, protein marker; lane 2, pellet of cell lysate; lane 3, pellet (inclusion body) of slow induction; lane 4, supernatant (soluble fraction) of slow induction with arrow showing hyper expressed cellulase protein

The slow induction of recombinant cellulase was attempted to achieve soluble expression by inducing the cells with 0.5 mM IPTG for the period of 16 h at 28 °C with shaking at 120 rpm. Cell lysate was prepared by mechanical bead lysis method and compared with previous insoluble protein pellet on 10% SDS-PAGE. As is evident in Fig. 5 (lane 4), most of the protein expression was visible in the soluble fraction of the cell lysate rather than inclusion bodies.

### Metal affinity purification of recombinant cellulase

**Fig. 6:**
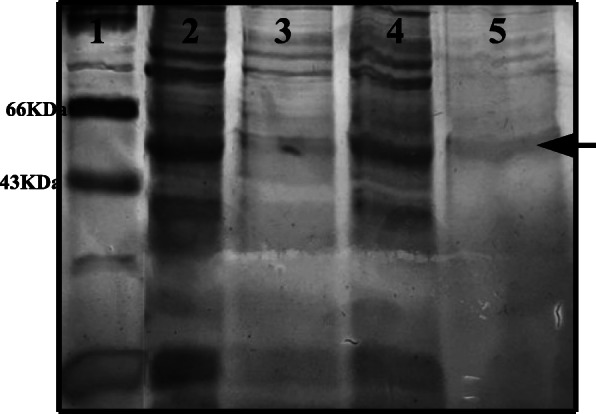
Protein profile of partially purified (metal affinity purification) recombinant cellulase on SDS-PAGE. Lane 1, protein marker; lane 2, supernatant of cell lysate; lane 3, flow through of supernatant; lane 4, wash fraction; lane 5, elution fraction (250 mM imidazole) with arrow showing cellulase band

The supernatant of cell lysate carrying recombinant cellulase with 6X His tag was loaded onto Ni-NTA affinity chromatography column. Purified elution fraction was loaded on 10% SDS-PAGE along with standard protein marker (medium range) to evaluate the purity of the protein. The gel was stained by silver stain. As is evident from Fig. 6, partial purification of recombinant cellulase, has been successfully achieved.

### DNS qualitative assay

**Fig. 7:**
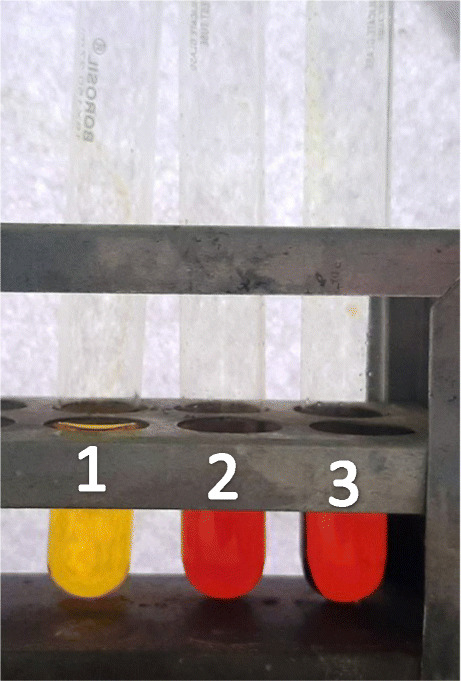
Qualitative analysis of reducing sugar by DNS assay. Tube 1, blank; tube 2, 1st elution fraction (250 mM imidazole); tube 3, crude supernatant of cell lysate

It is a colour-based assay for the detection of reducing sugar. As seen in Fig. 7, eluted fractions containing partially purified recombinant cellulase could digest the CMC substrate and release glucose molecules imparting orange colour post addition of DNS. This confirms that the soluble recombinant cellulase expressed in *E.coli* was active.

## Discussion

With the increasing importance of the industrial application of cellulase in a wide range of fields, researchers have been working for its large-scale production [1, 2, 13, 20, 21]. Bacterial and fungal sources are the potential options to produce an active enzyme. However, cellulases produced naturally by bacterial and fungal strains showed some limitations in terms of inoculum growth conditions, time and productivity of the fermentation process. Hence, recombinant cellulase has been explored as an alternative to overcome limitations of the naturally produced cellulases.

Source for the gene of interest and host for the protein production are the two fundamental criteria in recombinant protein/enzyme expression. Cellulases are composed of endoglucanase, cellobiohydrolase and β-glucosidase. Several reports are available for recombinant expression studies of these enzymes. In our work, BL21 (DE3) and pET21a (host—vector) combination was used for recombinant cellulase (endo-(1,4)-β-D-glucanase) production. Barros et al. [22] reported cloning and expression of cellulase gene from *R. flavefaciens* in HB101 strain of *E. coli*, cellulase gene was inserted within *Eco*RI site of pEcoR251 vector and expressed under *λ* promoter. In another study, randomly digested fragments of *C. firmi* genomic DNA was cloned in pBR322 and expressed in CSR603 strain of *E. coli* [23]. Cellulase gene from fungal source was studied by means of cDNA cloning and expression in λZAPII system by Gang-Ping Xue et al. [24]. Considering the upgradation in heterologous protein expression, researchers have changed the experimental strategies in later reports. Protease deficient bacterial and yeast cell lines and expression vectors with strong promoters were the most favoured combination in various studies [25].

Amongst fungal sources of cellulase gene, different strains of *Trichoderma* were widely studied. Zeng et al. [26] reported cloning and expression of endo-(1,4)-β-D-glucanase gene from *T. virens* and disulphide bond formation was achieved by using pET32a vector. In another study, Okada et al*.* [27], experimentally analysed the structural properties of recombinant EGIII protein (variant of endo-(1,4)-β-D-glucanase) using QM9414 strain of *T. reesei* as a source of cellulase gene. They have also mentioned comparative heterologous expression in bacterial and yeast systems with solubility as well as activity studies.

In the current study, *Bacillus subtilis* natto strain was selected as a source of cellulase (endo-(1,4)-β-D-glucanase) gene which was tested for cellulolytic activity by Congo red staining assay on CMC containing media. *Bacillus* is reported as the preferred genus for bacterial source of cellulase gene in most of the studies, although there are also reports for actinomycetes and other genera [17, 28]. Although it is known that *B. subtilis* (natto strain) produces multiple enzymes like amylase, cellulase and proteases, the cellulase enzyme from this strain is not much explored. Since this strain is mainly used for the fermentation of soybean products, its proteases are well characterised [16]. The cellulase gene from this strain was amplified to achieve the amplicon of 1500 bp, and it was further cloned in pET21a vector. Selected recombinant was transformed into BL21DE3 expression host and induced with 1 mM IPTG; the recombinant cellulase was observed in inclusion bodies. Expression of recombinant protein in inclusion bodies always poses a challenge during the purification step. It is hence always preferred to achieve the soluble expression of the recombinant protein which ensures less hassles during purification and also, it is likely to give active protein molecule..

Soluble expression of recombinant protein has the biggest advantage during the fermentation process as very few inherent bacterial proteins are expressed in soluble form. Additionally, soluble protein expression reduces downstream processing cost which has a direct impact on total production cost. There are several methods to convert proteins expressed in inclusion bodies into a soluble form, but it can be time consuming and expensive. Fusion of solubility tags to desired protein, selection of different vector-host combinations, and optimisation in expression conditions are the potential modification scientists attempt to achieve soluble protein expression. One such cost-effective approach reported by Munjal et al. [20], as recombinant extracellular cellulase expression in ethanologenic strain of *E. coli* by replacement of inducible promoter with native constitutive promoter (*gapA*). Variations in the usage of solubility tags/signal peptide are also reported by some researchers. Amore et al*.* [17] and Liu et al. [29] used signal peptide of source organism whereas Kim et al. [30] expressed cellulase on the outer membrane of *E. coli* in fusion with ice nucleation protein (membrane protein of *P. syringae*).

In the current work, we used the slow protein induction strategy with 0.5 mM IPTG induction for 16 h at 28 °C. This helped in achieving the cellulase expression in soluble fraction rather than inclusion bodies. This strategy was definitely advantageous as it helped in lesser downstream steps because no solubility tag was used. Solubility tag poses problems due to the requirement of its removal to obtain authentic protein. Slow induction process with a lesser concentration of inducer can reduce upstream process cost in large-scale production.

Increased solubility of cellulase protein and N terminal 6X His tag allowed simple two-step metal affinity purification of the protein [31]. Purity of the eluted fraction was evaluated in comparison with the crude cell lysate on SDS-PAGE. Partial purification of recombinant cellulase (55 KDa) was achieved. The recombinant enzyme was also found to be active by qualitative DNS assay. Standardisation for scaleup of fermentation process to achieve hyperexpression of soluble enzyme as well as the study of kinetic parameters of this soluble recombinant enzyme would be a prime objective of the further work.

## Conclusion

In the current work, recombinant cellulase enzyme was produced using cellulase gene of *B. subtilis* (natto strain) which is not much explored for its cellulase activity using standard shake flask fermentation protocol. Majority of the enzyme expression was seen in the inclusion bodies but when the fermentation parameters were changed for slow induction of protein, the cellulase expression was achieved in soluble fraction. Hence, optimisation of fermentation conditions attempted in this research work to achieve soluble expression of cellulase is the promising alternative. Future work would be required to evaluate if this strategy can be scalable as well as if it would be cost effective for large scale production.

## Data Availability

All the data generated and/or analysed during this study is included in this published article.

## Abbreviations

DNS: 3,5-dinitrosalicylic acid
Ni-NTA: Nickel-nitrilotriacetic acid
PCR: Polymerase chain reaction
LB: Luria Bertani
IPTG: Isopropyl β-D-1-thiogalactopyranoside
rpm: Revolutions per minute
SDS-PAGE: Sodium dodecyl sulphate polyacrylamide gel electrophoresis
CMC: Carboxymethyl cellulose
His: Histidine
bp: Base pairs
KDa: Kilodalton
